# The Treatment of Pellagra

**Published:** 1916-10

**Authors:** Duane Meridith

**Affiliations:** Professor of Embryology, Histology and Research Medicine, Medical Department of T. C. U. University. Fort Worth, Texas


### The Treatment of Pellagra.

BY DUANE MERIDITH, M. D., PROFESSOR OF EMBRYOLOGY, HISTOLOGY AND RESEARCH MEDICINE, MEDICAL DEPARTMENT OF T. C. U. UNIVERSITY. FORT WORTH, TEXAS.

In presenting this paper to the medical profession I realize the enormous amount of literature that is being written upon this subject, and to use a slang expression, "without anybody getting anywhere." It is with the idea that what I have to add will be of some help to the doctor out in the country who is trying to get his patients well and is not so much concerned as to how the disease is transmitted, or whether it is due to cornbread or the lack of vitamins, that I prepare this paper.

The history of this disease is too well known to the medical world for me to mention it here.

The etiology of this disease is still a disputed point. Some think that it is due to the lack of vitamins (Goldberger's theory). A great many others think it is due to some infecting organism. Personally, I belong to the last group, as I have been able to isolate an organism that belongs to the higher bacteria which fulfill Koch's laws in many ways, and I hope within the next year to prove that it is the true cause and that even the most skeptical will admit its identity.

I think one of the first essential requirements in the treatment of this disease is the recognition of the early symptoms. This is the prodromal stage as it were. This is especially true of the disease in adults who may have the disease for years without giving more than one of the cardinal symptoms of diarrhea, skin eruption or nervous symptoms. Each of the cardinal lesions has a great number of sub-lesions that are not exactly typical. For instance, a patient that gives no history of any skin lesion, but has marked diarrhea and nervous symptoms, will show a pustular eruption in the hair, and if you will watch each patient you will observe that he is always rubbing his head. Sometimes this eruption is nothing more than a papular eruption that occurs along the edge of the hair, especially of the forehead, or it may occur on the lower limbs. It may occur as a severe case of urticaria that will leave pigmented spots. In a few cases I have seen the pustular form of eruption on nearly the entire body, and were it not for other symptoms one would never suspect the patient of having pellagra.

The gastro-intestinal tract has even more atypical symptoms that one has to be on the watch for, such as a burning sensation of the nasal orifice, lips and tongue, with a chronic sore throat that does not yield to local treatment and which may extend down the oesophagus and affect the stomach when the patient will complain of indigestion with sour stomach, and on belching, say that it takes the skin off the whole of the oesophagus, leaving it burning and raw. A patient with this condition usually has no other symptoms outside of a general tired feeling, and upon doing any active work will become very weak and fainty and usually complains of turning blind when stooping over. Upon close inquiry of his bowel actions, you will find that he has occasional spells of diarrhea, and if he is the least bit constipated, will have grassy green stools at some period of his sickness. They all have more or less gas in their bowels and they often come to you complaining of their abdomen swelling and want something for relief.

As the intestinal tract seems to be the favorite haunt of the causative factor, one often meets with trouble in the adjoining organs, such as acute choleocystitis and appendicitis due to the pellagra organism. When you operate you find very little pathology compared to the symptoms complained of by the patient, as the appendix will appear nearly normal as will also the gall bladder, and the unfortunate part is that you do not cure your patient. Another frequent site of pellagra lesions is the bladder, and in women some of the female organs with most frequenty some displacement or falling of the womb with continued metrorrhagia or severe neuralgic pains in the ovaries and tubes and which I think often gives rise to cystic ovaries. I am sure that there are many women going to the operating table for some pelvic trouble that could be easily cured if their doctor would take the time to work out their cases carefully.

Of the nervous symptoms, burning of the end of the fingers has been one of my most frequent complaints, with a general weakness upon any active exertion, dull headache, particularly in the back of the head and neck, and sleeplessness at night, and general neuralgic pains in the body. One of the most frequent sites for these pains is along the lower border of the ribs and this is especially true of the left side and in the left groin and hip.

I have seen these pains so severe in the groins that the patient could not turn over in bed or walk, and when this occurred on the right side, appendicitis and renal colic have been diagnosed by other good physicians.

Another symptom of the disease is hysterical spells and sometimes the patients have distinct convulsions. I have had four cases of the last type within the past year. None of these cases had skin lesions when the diagnosis was first made, but two have since developed typical lesions.

From the above rambling description of the minor symptoms of this disease, one can see why a very carefully prepared history is of so much importance in the early diagnosis of this disease. There is nothing else outside of a white blood and differential count that gives us any information. An early diagnosis is one of the means of successfully treating this disease, as these patients yield to treatment very readily in the early stages. If you should take away quinine from the medical profession I had rather treat pellagra than malaria.

I shall now take up the treatment, which is largely dietetic, hygienic and medicinal. Dr. Goldberger has given us a diet list that is very good indeed, in which he advises that the food consist principally of protein. I have followed this list very carefully for the past year and have found it good, but I find that my patients do better by feeding them oftener than he does. I give my patients their regular fare but have them drink a glass of the following mixture between meals and on retiring at night: Add four raw eggs to a quart of sweet milk and to this add a dram of dilute hydrochloric acid. This mixture is to be well beaten and placed upon ice and used when needed. I find that my patients do well upon this as it does not upset their stomachs and along with their regular diet will gain rapidly in weight.

Hygienic. I think that the same precautions should be used as are used in the treatment of tuberculosis, i. e., that the patients should have a room to themselves and their drinking and eating vessels should be kept separate from other members of the family. While I believe that one of the biting flies is responsible for the transmission of the disease in the majority of the cases, I readily see how it might be transferred by using the same drinking vessel if the patient had an active mouth lesion which they all have; and the spore of the organism lodging in the intestinal tract might find suitable grounds for its development, for the organism that

I have been working with is a facultative anarobe. I try to keep my pellagrins out of the sun as much as possible and in order to do this put them to bed—that is where they have the disease with any severity, and also by putting them to bed their strength is conserved. These patients do better to have a warm bath each day.

To successfully treat these people one has to be an optimist of the extreme order and a very successful liar, as I find that it is best not to tell the patients what is the matter with them. You may tell a relative, but if possible keep the true cause from the patient, as they usually get to brooding over their trouble and it will take just three times as long to get the same result as from those who do not know.

The treatment of this disease is still largely empirical. From my study of the disease and the organism, I think that the lesions of this disease are entirely due to this organism, just as the lesions in measles and scarlet fever and the other acute infectious diseases we know to be due to the organisms that cause the disease. I think that the organism of pellagra lives off the waste products of epithelial cells and that the symptoms of the disease are due to the constant destruction of these organisms within the blood with the liberation of a toxaphore group and that a ferment is produced that is responsible for the destruction of the organism, a true anaphylasis phenomena.

In this, as in all diseases of this character, arsenic is one of our main drugs, and it is surprising the amount of arsenic one of these patients will stand without giving any symptoms of the drug. If the patient is well to do and can pay his bills, I usually give cacodylate of sodium, as I have never seen any bad effect from this drug outside of some very sore arms. The one drug that has given me the best result, that is, if the patient can stand the mercury, is Donovan's Solution. In my hand when given early, especially to those who have the skin lesions, the latter clear up remarkably well in a short time, and as most of our pellagra cases occur in the poor, it is an inexpensive drug that anyone can afford. I think our arsenic preparations give their best results in the skin and nervous type. In some of my acute cases that had a severe neuritis and fever, I have given 20 drops of Donovan's Solution every three hours to relieve the pain. I have found that this drug would do for the patient what morphine would not do—relieve his pain and at the same time cure his disease.

It is in the intestinal type of this disease that we have the most stubborn cases. I have used every drug that was ever recommended for this disease in this type of cases. Thymol does some good when given in four-grain doses every four hours; so does picric acid in 1 per cent solution given in teaspoonful doses. I have had my best results from 8 per cent argyrol in nuclien solution given in teaspoonful doses every three hours, or 5 minims of ichthyol capsules given the same way. If one will give a c.c. of 10 per cent sodium citrate hypodermically each day to neutralize the blood ferment, one will get much better results from the treatment. If the patient cannot come to the office, have them drink a glass of water that has had added to it a half teaspoonful of baking soda. Bismuth is another drug that when given in large doses gives some results where the diarrhea is bad. I have found that a month's use of one particular drug usually wore out its effect, so that a constant changing of medicine is essential to keep the patient improving. The drug that has given me my best results has been Donovan's Solution, and to get the effect from it one has to give it in sufficient sized doses so as to keep a slight puffy condition under the eyes. I have frequently examined the urine of my patients who are taking large doses of arsenic, and have found no evidence of kidney disturbance either chemically or microscopically.

I have had but a few patients who could not take Donovan's Solution, and in these I have used everything, but found that I could rely upon ichthyol, argyrol and sodium cacodylate better than upon any other drugs. But above all things watch your patient and meet the symptoms when they arise, and feed your patient to the limit, and then by keeping him in bed and giving one of the above mentioned drugs by first trying the patient on a small dose and gradually increasing the dose as it is indicated, and if you see that there is no improvement by changing to some other drug, I think that very few cases of pellagra would be lost.

---

In comminuted fractures, the best method is the fixation of main fragments, leaving small pieces in the best possible position between the major fragments.—*H. F. Derge, Wisconsin Medical Journal.*

---

Off His Fee.—Doctor: You are suffering from a complication of diseases—at least six.

Patient: I suppose you'll allow me a discount on half a dozen, doctor?—*Carrizo Springs Javelin.*

